# Life History Transitions at the Origins of Agriculture: A Model for Understanding How Niche Construction Impacts Human Growth, Demography and Health

**DOI:** 10.3389/fendo.2020.00325

**Published:** 2020-05-21

**Authors:** Jonathan C. K. Wells, Jay T. Stock

**Affiliations:** ^1^Childhood Nutrition Research Centre, Population, Policy and Practice Programme, UCL Great Ormond Street Institute of Child Health, London, United Kingdom; ^2^Department of Anthropology, University of Western Ontario, London, ON, Canada; ^3^Department of Archaeology, Max Planck Institute for the Science of Human History, Jena, Germany

**Keywords:** life history theory, origins of agriculture, population growth, niche construction, nutrition transition, diet, infectious disease, trade-off

## Abstract

Over recent millennia, human populations have regularly reconstructed their subsistence niches, changing both how they obtain food and the conditions in which they live. For example, over the last 12,000 years the vast majority of human populations shifted from foraging to practicing different forms of agriculture. The shift to farming is widely understood to have impacted several aspects of human demography and biology, including mortality risk, population growth, adult body size, and physical markers of health. However, these trends have not been integrated within an over-arching conceptual framework, and there is poor understanding of why populations tended to increase in population size during periods when markers of health deteriorated. Here, we offer a novel conceptual approach based on evolutionary life history theory. This theory assumes that energy availability is finite and must be allocated in competition between the functions of maintenance, growth, reproduction, and defence. In any given environment, and at any given stage during the life-course, natural selection favours energy allocation strategies that maximise fitness. We argue that the origins of agriculture involved profound transformations in human life history strategies, impacting both the availability of energy and the way that it was allocated between life history functions in the body. Although overall energy supply increased, the diet composition changed, while sedentary populations were challenged by new infectious burdens. We propose that this composite new ecological niche favoured increased energy allocation to defence (immune function) and reproduction, thus reducing the allocation to growth and maintenance. We review evidence in support of this hypothesis and highlight how further work could address both heterogeneity and specific aspects of the origins of agriculture in more detail. Our approach can be applied to many other transformations of the human subsistence niche, and can shed new light on the way that health, height, life expectancy, and fertility patterns are changing in association with globalization and nutrition transition.

## Introduction

Over recent millennia, human populations have regularly reconstructed their own subsistence niches, a practice known as “niche construction” (1). Arguably the most important such transformation occurred with the origins of agriculture. From around 20,000 years ago in the Levant, for example, populations began to aggregate in long-term settlements, and to systematically exploit wild grain (2) and produce new staple foods such as bread (3), which led to widespread domestication of plants and animals throughout the Near East (4). Over the past 10,000 years, the domestication of numerous species of plants and animals has occurred independently and in different ways in different parts of the world (5, 6), though a small proportion of humanity continues to practice hunting and gathering. Such domestication events also led to increased use of secondary animal products such as milk, which further led to the independent evolution of lactase persistence in some human populations (7). However, the adoption of agriculture is only one such example of niche construction. We can use the same conceptual approach to consider more recent societal transformations, such as industrialisation, or globalization and the ongoing nutrition transition. These transformations of the human niche are widely understood to generate both benefits and costs for human health.

Many of these transitions have been sufficiently rapid that the biological consequences cannot be attributed only, or even primarily, to genetic change. Rather, physiological and behavioural plasticity are also implicated. Various mechanisms of developmental plasticity are now understood to contribute to substantial variability in phenotype and health outcomes through the life-course. For example, variability in nutrition, growth rates, and exposure to infections in early life shapes many traits at later ages, including body size and composition, reproductive scheduling, and the risk of various diseases (8). The transitions associated with the origins of agriculture, and the domestication of animals and use of secondary animal products, were both transitions in the energetics of the human diet, where dietary shifts were characterized by more energetically-rich but less diverse sources of food and increased risk of famine. However, these subsistence shifts also involved more fundamental transformation of the human niche, for example by changing patterns of physical activity and reshaping exposure to predators and pathogens and social inequality (9).

Today, we face a paradox that apparent improvements in human living conditions, including economic growth and nutrition transition, are strongly associated with emerging epidemics of chronic non-communicable disease, such as obesity and cardiovascular disease (10). Moreover, while the burden of infection appeared to decline over the twentieth century (11) through the development of diverse forms of prevention and medical treatment, many pathogens are evolving resistance to drug therapies while new diseases can evolve (12, 13). The burden of infection faced by future human populations may therefore be more threatening, and there is an urgent need to understand how alterations to human subsistence niches impact our biology and health.

Here, we develop a conceptual framework based on evolutionary life history theory (14), and apply it to improve understanding of how human biology changed in ancestral populations in association with the origins of agriculture. In this article, we use this term to refer to the suite of domestication events of plants and animals that is highly variable temporally and geographically, but which fundamentally changed the human subsistence niche wherever it occurred. By focusing on patterns of change that occurred in a major past transformation of our subsistence niche, we may gain valuable new insight into what is happening in contemporary populations. The patterns of change that we describe are largely regulated by hormonal mechanisms and many occur during development, hence our framework offers a new perspective on the role of endocrinology, in particular pediatric endocrinology, in the evolutionary trajectory of our species.

It has long been recognised that the emergence of agriculture had profound effects on human biology, at the level of both populations and individuals. For example, the shift from foraging to farming was associated with major increases in population size in some places, demonstrated by the emergence of villages and urban settlements from 12,000 to 5,000 years before present (BP) in the Levant, China, India, and West Africa (5). Population growth in the pre-agricultural Palaeolithic is likely to have occurred at a very slow overall rate, subject to local boom-bust dynamics (15). In contrast, the transition to agriculture was associated with more systematic population growth (16, 17).

Exactly what stimulated the adoption of agriculture is controversial. Boserup (18) and Cohen (19) suggested that larger populations stimulated a need for agricultural production to meet food requirements. The main demographic change was not a reduction of mortality, but rather a decrease in the average inter-birth interval, so that any increases in mortality were over-compensated by rising fertility (16). However, a classic review of the literature by Cohen and Armelagos found many indications that health deteriorated in the early agricultural era (20). This perspective—that human populations expanded in size, despite living conditions actually worsening (20)—has become the dominant paradigm, however little attention has been directed to whether these parallel trends might have some deeper biological link.

In this review, we develop a new hypothesis to explain these trends: that the correlated changes in phenotype and population size reflect a reorganization of human life history strategy, to accommodate the composite change in ecological conditions provoked by niche construction (10). Changes in each of food supply and environmental risk are expected to impact life history strategy, especially when both factors change simultaneously. We first describe life history theory and summarise evidence for trade-offs between individual life history traits obtained from studies of contemporary human populations. We then consider how the onset of agriculture altered the human niche, impacting a series of selective pressures including energy supply, dietary diversity, and pathogen burden. We review evidence for life history trade-offs in the archaeological record, noting that these shifts are likely to have been variable and distributed over a range of timescales, depending on how the transition to agriculture played out locally. Finally, we discuss how, if our hypothesis is correct, it may apply to other systematic shifts in living conditions that had an impact on human energetic ecology, such as industrialisation.

## Life History Theory and Phenotypic Change

Life history theory offers unique opportunities for biologists to investigate phenotypic change in populations over time (14). The value of this theory is 2-fold—first, it models variability in phenotype in general, rather than individual traits, and second, it can address phenotypic variability or change that arises both through genetic adaptation, and also through mechanisms of plasticity, whether physiological, developmental, or behavioural (21).

Life history theory considers how organisms maximise their genetic fitness through harvesting resources from the environment, and investing them in a suite of biological functions throughout the life-course (14, 22). In theory, multiple currencies of resource allocation may be important, such as different nutrients (23), but in practice the theory gives priority to “energy” and “time” as the most important resources, and assumes that organisms making the best use of energy over their lifespan will receive the highest fitness payoffs (24).

The theory assumes that for any individual organism, the supply of energy is finite, and that allocating more energy to one function precludes its allocation to other functions (22). Traditionally, life history theorists focused on three competing functions, namely maintenance (M), growth (G), and reproduction (R) (22). Maintenance refers to keeping the body in good condition through diverse homeostatic process, thereby promoting longevity and maximising the future opportunities for reproduction. Growth refers to the process of development and maturation, and typically occurs prior to reproduction in most mammals. Reproduction refers to all processes involved in finding a mate, producing offspring and investing in them, and essentially allocates energy to the next generation. From an inclusive fitness perspective, investment in “reproduction” may incorporate patterns of social behaviour that benefit kin who share genes (25).

The principle of competition between these functions results in energy-allocation trade-offs between them at any given stage of life. Natural selection then favours the emergence of life history traits, and broader developmental or life-course strategies, that are shaped by such trade-offs. Each organism's life history can be summarized as a cumulative series of energy-allocation decisions, represented by a suite of developmental and reproductive traits. These include how fast and large to grow, how to address risks and defend against threats, and how to schedule reproductive effort (14).

**Figure 1:**
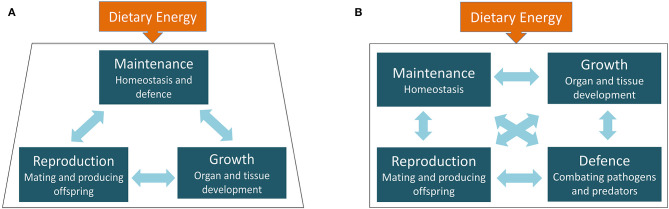
The principle of life history theory, showing **(A)** the traditional 3-function model and **(B)** our expanded 4-function model. The arrows represent the binary trade-offs, between maintenance, growth and reproduction in the traditional model, and between maintenance, growth, reproduction, and defence in the expanded model.

In practice, however, we have argued that it is more appropriate that four life history functions be differentiated (21). Whilst “defence” (D) against pathogens and predators was initially considered to come under the general umbrella of maintenance (22), it is increasingly recognised that defence is subject to overt trade-offs against each of maintenance, growth, and reproduction (10). Both immune function and activating the “fight-or-flight” response to avoid predation reduce the availability of resources for other life history functions. In Box 1 and Figure 1, we review the implications for life history theory of treating defence as a separate life history function, increasing the number of binary trade-offs that can be tested in empirical work.

Box 1 **Incorporating defence as a separate function into life history theory.**

Early work on life history theory considered that there were three competing functions (maintenance, growth, and reproduction) giving rise to three potential binary trade-offs (14, 22), as illustrated In Figure 1A. Particular attention was directed to the trade-off between reproduction and survival, whereby producing more offspring was assumed to reduce investment in homeostatic maintenance (e.g., through mechanisms such as oxidative stress), thereby accelerating ageing and shortening parental lifespan (26). For example, experimental studies in animals tested the effect of imposing a greater reproductive burden (e.g., augmenting brood size in birds) on parental lifespan (27), while observational analyses in humans tested for inverse correlations between fertility and lifespan (28, 29).

We propose that defence can be differentiated conceptually from maintenance as involving metabolic responses that respond to the activities of external organisms that threaten survival or fitness through predation or infection/parasitism. On this basis, defence manifests specifically as short-term responses to combat these external threats, and to repair any immediate damage to organs and tissue, with these responses necessarily precluding optimal investment in other life history functions. In contrast, the routine allocation of resources to preserving organs, tissues and immune function in good operating condition, in the absence of specific activities by predators, pathogens, or parasites, can be considered homeostatic maintenance.

Treating defence as a discrete life history function increases the number of binary trade-offs in the model from three to six, as illustrated in Figure 1B. This approach offers a richer conceptual framework for investigating adaptation to ecological conditions or change (note that the number of binary trade-offs can be further expanded by considering those across generations, as illustrated in Figure 4). We suggest that the value of this framework may be further enhanced by paying particular attention to trade-offs that manifest during development, as well as those occurring during adult life. For example, many of the most salient markers of growth (e.g., limb lengths) reach their final value at the start of adult life, meaning that the most important trade-offs involving these outcomes must have occurred during earlier stages of development. It has already been recognised that the effect of mortality risk on life history trade-offs varies according to the age of the organism (30), and we suggest that the same issue is relevant for growth, which for example has relatively high costs in infancy and adolescence but reduced costs during childhood and much lower costs during adult life (31).

In conventional life history theory, much attention has been directed to “extrinsic mortality risk” as a key factor shaping the likelihood of survival and lifespan. For example, the “disposable soma” theory assumes that the higher the risk of mortality, the lower the optimal level of investment in maintenance as the pay-offs are unlikely to be recouped (32). This approach expects an inverse association between mortality risk and lifespan. However, by differentiating defence as a discrete function, we can see that threats to survival and fitness can be countered by mounting specific responses to reduce the risk of mortality, but at a cost to the ability to invest in other functions. Not all infections directly threaten survival, but they can still demand expensive immune responses. Paradoxically, this scenario results in the potential to observe positive correlations between lifespan and markers of ill-health, as individuals manage to survive for longer, but in sub-optimal condition.

Initially, life history theory was primarily used to explore phenotypic differences between species (30). The diverse selective pressures associated with any given ecological niche favour the emergence of broad species-specific energy allocation strategies, underpinned by genetic adaptation. Life history variability is assessed by considering a set of demographic and physical traits that can be readily assessed in any organism. For mammals, these traits include size at birth, time taken to reach maturity, the frequency of reproducing, the number of offspring produced per reproductive event, and the total lifespan (30).

The two main ecological factors driving life history trade-offs across species are the supply of resources (effectively, energy), and the risk of mortality (33). First, organisms subject to high mortality risk are unlikely to maximise fitness if they prolong the period of growth, instead selection favours earlier reproduction. Moreover, because of the high risk of mortality for each individual offspring, organisms in such environments should produce large numbers of offspring but allocate little parental investment to each. In this way, mortality risk inherently shapes life history traits such as physical growth, maturation rate, and reproductive scheduling (30, 32). Second, all other things being equal, a greater supply of energy allows individual organisms to grow bigger, or the number of offspring produced to be greater, or the investment per offspring to be increased, promoting offspring fitness. Again, therefore, local ecological productivity shapes life history traits.

Within species, genetic variability may also contribute to life history variability among individuals. For example, most life history traits in humans have been shown to have a component of genetic variability, demonstrated at the broader level by calculations of heritability and at more specific levels by the findings of genome-wide association studies (Table 1) (53).

**Table 1:** Evidence for heritability of life history traits and examples of individual genetic determinants.

**Trait**	**Population**	**Heritability**	**GWA evidence**	**References**
Birth weight	UK twins	44%		(34)
	Norwegian families	31%		(35)
	Swedish twin pairs	25–40%		(36)
	Meta-analysis of 69,308 Europeans from 43 studies		7 alleles associated with birth weight variability	(37)
Age at Menarche	Australian sister-pairs	69%		(38)
	Dutch families	70%		(39)
	US families (Fels study)	49%		(40)
	Meta-analysis of 182,416 women of European descent from 57 studies		106 alleles associated with variability in age at menarche	(41)
Adult height	Gambian families	60%		(42)
	Indian families	74%		(43)
	European twins	81%		(44)
	~450,000 UK Biobank participants of European ancestry		3,290 near-independent SNPs associated with variability in height	(45)
Body mass index	Finnish twins	80%		(46)
	Nigerian families	46%		(47)
	Chinese twins	61%		(48)
	~450,000 UK Biobank participants of European ancestry		716 near-independent SNPs associated with BMI	(45)
Age at menopause	US families (Framingham)	52%		(49)
	Dutch mother-daughter pairs	44%		(50)
	Dutch twins	71%		(51)
	17,438 women from two US cohorts		13 SNPs associated with variability in age at menopause	(52)

In non-human animals, experimental support for the notion that natural selection shapes life history traits has been provided by elegant studies of small freshwater fish called guppies, living in the mountain streams of Trinidad (54). These studies clearly illustrate the influence of mortality risk on life history strategy. Typically, the streams have waterfalls that restrict predators to the lower reaches. Guppies living downstream, with a high risk of predation, grow faster, and start to breed earlier than those living upstream. Transplanting downstream guppies into the upstream environment resulted in a slower life history emerging across generations—the onset of reproduction was later, and fewer but larger offspring were produced. In contrast, introducing the predators upstream elicited a faster guppy life history strategy, indicated by earlier onset of reproduction. Further studies have shown that this variability is in part genetic, supporting the hypothesis that different life history strategies can evolve through genetic change in different environments (54).

Similar to work on other species, much research on human life history strategy has analysed the same set of demographic traits, i.e., size at birth, growth and maturation rates, adult size, reproductive scheduling, and lifespan (55–57). However, a range of somatic traits can also be considered from the same perspective. The “embodied capital” conceptual model of Kaplan and colleagues considers the body in terms of a range of traits that reflect somatic investment (58). This investment may be considered in physical terms, expressed through the characteristics of individual tissues and organs, or in functional terms, expressed through a range of capabilities. Of particular relevance for studying past human populations, this conceptual approach allows the life history framework to be applied to many aspects of human anatomy, physiology, and morphology.

For example, adult stature is a marker of investment in overall growth, adipose tissue is a marker of investment in reproduction for females (59), and in defence (for funding immune function) for both sexes (60), while organ mass and quality are markers of investment in maintenance (61). This means that variability across different morphological traits can be used to index life history trade-offs, offering a new perspective on the archaeological skeletal record.

In stochastic environments, however, there are benefits to withholding a portion of energy from immediate investment, to be able to draw on it at some future time when new stresses or opportunities emerge. Several different strategies are available whereby organisms may store energy in generalised forms, so that it can be allocated to any life history function when needed (62). The origins of agriculture led to food surpluses and storage (63), while the origins of dairying involved the use of secondary animal products that provide a constant source of energy rich food, as grazing animals process grasses that humans cannot eat into milk and its by-products. Beyond the physical storage of foodstuffs, there are other social and biological means of storing energy. Mutually supportive social relationships are one such method, for example humans are “cooperative breeders,” whereby kin provide support to mothers during reproduction and mitigate some of the energetic costs (62). A second method is the storage of energy as lipid in adipose tissue. Should dietary energy intake decrease unexpectedly, or infection elicit an immune response, energy needs can be met by oxidising lipid stores (62). Similarly, humans are “capital breeders,” whereby females tend to store energy prior to pregnancy so that reproduction is viable regardless of external ecological conditions (64). As a fundamentally social species that also has greater levels of body fat than most other primates, humans have evolved the capacity to store energy in several different forms, indicating that our life history strategy was strongly shaped by stochastic environments (65).

So far, we have considered how human life history traits in general may have emerged through genetic adaptation in response to variable ecological conditions. However, the same traits also show substantial plasticity, indicating that such responses may also occur over faster timeframes. Here, selection has favoured the evolution of reaction norms that allow fitness-maximizing traits to emerge in response to stimuli and stresses encountered within the life-course. Reaction norms refer to the spectrum of phenotypes produced by a genotype across a range of environmental conditions (14). To highlight this plasticity, Table 2 summarises secular trends in human life history traits, indicating their capacity to respond to changing ecological conditions and generate new trade-offs.

**Table 2:** Evidence for plasticity in life history traits, demonstrated by secular trends.

**Trait (units)**	**Population**	**Rate in units per decade**	**Decade per SD change**	**References**
Birth weight (g)	Canada (1985–1998)	27.7	18.1	(66)
	Norway (1967–1998)	36.8	13.6	(67)
	India (1963–1986)	32.2	15.5	(68)
	Papua New Guinea (1969–1996)	70.4	7.1	(69)
	Vietnam (rural) (1999–2010)	95.0	5.3	(70)
Age at menarche (y)	Spain (1925–1962)	0.26	3.8	(71)
	South Africa (black) (1956–2004)	0.50	2.0	(72)
	India (1979–2003)^*^	0.20	4.9	(73)
	Korea (1920–1986)	−0.64	1.6	(74)
	Colombia (1944–1984)	−0.55	1.8	(75)
Height (cm)	Czech (f) (1935–1955)^$^^*^	1.1	5.4	(76)
	Indian (f) (1979–2003)^*^	2.2	2.1	(73)
	Portugal (m) (1904–1996)	1.0	6.1	(77)
	Poland (1965–1995)	2.1	2.9	(78)
	Belgium (1830–1980)^$^	1.0	6.0	(78)
Adult BMI (kg/m^2^)	Sweden (f) (1985–2002)	1.2	2.5	(79)
	Greece (m) (1990–2006)	0.6	5.3	(80)
	United States (m) (1980–1987)	0.8	3.7	(81)
	China (1991–2011)	1.2	3.0	(82)
	Brazil (f) (1975–2003)	1.1	2.7	(83)
Age at menopause (y)	Spain (1883–1941)^$^	0.34	11.7	(84)
	Sweden (1908–1930)^$^	1.00	4.0	(85)
	United States (1912–1969)^$^	0.59	6.8	(86)
	Iran (1930–1960)^$^	0.70	5.7	(87)
	Korea (1927–1947)^$^	1.7	2.3	(88)

*BMI, Body mass index; m, male; f, female*.

* *Based on mothers being on average 24 years older than daughters, and assuming that the data were collected the year prior to publication*.

$ *Period over which trend assessed based on age at birth*.

Beyond any genetic determinants, therefore, life history strategies may vary through mechanisms of developmental plasticity, through which phenotype may be adjusted in association with recent or prevailing conditions. Such phenotypic adjustments can then be considered to have adaptive benefits, promoting survival and fitness. For example, secular declines in mortality risk are associated with secular increases in adult height (89), indicating that in benign environments, energy can be re-allocated from defence to growth. Similarly, patterns of growth in early life predict the timing of pubertal maturation, though in different ways depending on the quality of the environment (33).

Overall, life history strategies can change over time through both genetic and plastic responses, and both mechanisms may be relevant to phenotypic change associated with the origins of agriculture. Regardless of mechanism, such changes in trade-offs are assumed to be fitness-enhancing. Moreover, this theory predicts fundamental connections between changes in different biological traits. We emphasise that both natural selection, and ecological stresses within the life-course, do not act on individual traits, rather they act on strategies (90), which can be readily conceptualised as trade-offs. For example, we should focus not on height as a discrete outcome, nor even on the *strategy* of growing, but rather on the *trade-off* between allocating resources to growth vs. other life history functions. Our argument is that the origins of agriculture provoked trends in many components of biology, such as body size, fertility, and health status, through shifting these trade-offs to new niche-specific optima. To provide empirical support for this theoretical framework, we now review evidence for life history trade-offs in contemporary human populations, focusing primarily on plastic responses.

## Evidence for Life-History Trade-Offs in Humans

**Figure 2:**
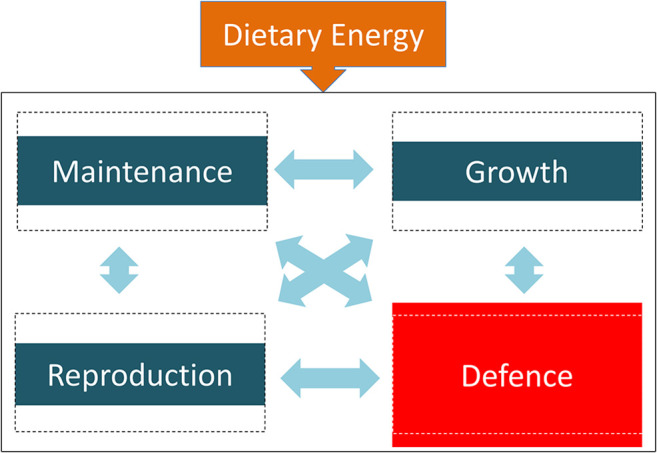
A life history trade-off, whereby allocating more energy to defence (e.g., from fighting an infection) results in less energy being available for maintenance, growth, and reproduction. The dotted line boxes indicate “equal” levels of investment across the four functions, and the coloured boxes indicate the actual magnitude of investment.

Many studies illustrate trade-offs between life history functions, though the findings are often not presented within this conceptual framework. Trade-offs might be driven by variability either in energy supply, or in the energy demanded by particular biological functions. In each case, the optimal allocation of energy between competing functions may change. For example, Figure 2 illustrates how an infection may elicit increased energy allocation to immune function, at a cost to all three other functions. In practice, most studies enable only two-function (binary) trade-offs to be considered. Between the four life history functions that we propose, a total of six binary trade-offs can be assessed. Evidence for each of these is now briefly reviewed, addressing where possible both short-term trade-offs that may be reversible (evident for example in adults) and also developmental trade-offs in early life that may be less reversible. Specific examples are also summarised in Table 3.

**Table 3:** Evidence for life history trade-offs in humans between maintenance (M), growth (G), reproduction (R), and Defence (D).

**Trade-off**	**Population**	**Life-history trait**	**Exposure**	**Outcome**	**References**
M-G	UK	Longevity	Rapid infant growth	Arterial stiffness	(91)
	UK	Longevity	Post-natal growth	High blood pressure	(92)
	India	Cellular health	Adult weight gain	Telomere attrition	(93)
	US	Cellular health	Adult weight gain	Telomere attrition	(94)
M-R	Global data	Maternal longevity	Reproductive effort	Shorter lifespan	(95)
	UK	Maternal longevity	Reproductive effort	Shorter lifespan	(29)
	Finland	Maternal longevity	Bearing twins	Shorter lifespan	(96)
	UK	Bone health	Early menarche	Reduced bone strength	(97)
M-D	Europe	Longevity	Infant disease load	Shorter adult lifespan	(89)
	Ethiopia	Mental health	Fetal fat deposition	Poorer mental health	(98)
	Meta-analysis	Metabolic homeostasis	Hepatitis C infection	Increased risk of type 2 diabetes	(99)
	Meta-analysis	Metabolic homeostasis	Periodontal disease	Increased risk of cardiovascular disease	(100)
G-R	SS Africa	Child growth	Number of siblings	Growth declines w. sibling no.	(101)
	UK	Child growth	Earlier reproduction	Low birth weight	(102)
	UK	Bone health	Early menarche	Reduced bone size	(97)
	India	Adult height	Early menarche	Short adult stature	(103)
G-D	SS Africa	Child growth	Maternal malaria	Low birth weight offspring	(104)
	Guatemala	Child growth	Diarrhoeal disease	Reduced growth	(105)
	Ecuador	Child growth	Immune activity	Reduced growth	(106)
	Europe	Adult height	Infant mortality rate	Mortality declines predict taller height	(89)
R-D	Senegal	Maternal mortality	Malaria	Reproduction increases infection risk	(107)
	UK athletes	Reproductive investment	Endurance exercise	Decline in testosterone	(108)
	UK athletes	Immune function	Endurance exercise	Increase in immune markers	(108)
	Malaysia	Reproductive investment	Reduced maternal stress	Increased breast-milk transfer	(109)

*SS Africa, sub-Saharan Africa*.

### Maintenance-Growth (M-G)

By strict definition, a trade-off between maintenance and growth can only occur during development, as growth in its normal sense ceases with the realisation of adult size. However, a looser definition of growth, which extends to tissue deposition and renewal processes, allows adult phenotype to be addressed. For example, bone maintenance continues through adult life and may be adversely affected by dietary or infectious stresses, as well as by reproduction in women (110). Similarly, adult weight gain, which comprises both fat and lean tissue, is associated with faster telomere attrition, a marker of cellular aging (94).

During development, reduced energy supply affects tissues to different degrees, as recognised by the thrifty phenotype hypothesis (111). Essential organs such as the brain and lungs are protected, at a cost to other organs (112, 113). In particular, the brain has an obligatory demand for energy, and meeting this demand can directly impact on the growth of competing tissues, such as the liver, pancreas, and muscle mass, which contribute to metabolic homeostasis (114). In turn, the preservation of homeostatic capacity slows the rate of ageing and promotes longevity.

In a study comparing lowland and highland children from Peru, for example, highland children exposed to high composite levels of ecological stress (poverty, under-nutrition, hypoxia, infections) protected growth of the brain and torso, at a cost to limb lengths, in particular the length of the tibia (112). Similarly, survivors of severe-acute malnutrition in Malawi protected both their brain and their lung function (essential for supplying the brain with oxygen) in mid-childhood, at a cost to leg length and muscle function (113). In turn, leg length is a strong predictor of metabolic health in adult life (115, 116). Thus, when energy supply is restricted, protecting brain growth comes at a direct cost of a reduced capacity for maintenance, which may contribute to an increased risk of chronic diseases at later ages (111, 117).

Although maintenance is usually measured at the level of physiological homeostasis, physical activity level can also be considered as a broader marker, though it is also relevant to other life history functions. At a behavioural level, activity is a key aspect of subsistence effort (118) but it also contributes to cellular homeostasis, promoting antioxidant enzymes that scavenge free radicals and prevent telomere attrition (119–121). Among a rural population of Yucatan Maya, where children used to provide significant levels of domestic and subsistence labour to the household economy, a longitudinal analysis showed that those demonstrating greater allocation of energy to physical activity were shorter and lighter than their less active peers (122). However, as we discuss below, physical activity also plays a unique role in life history trade-offs, as cooperative behaviour and “labour subsidies” allow the maintenance needs of some individuals to be met by the physical activity patterns of others (118).

### Maintenance-Reproduction (M-R)

A trade-off between maintenance and reproduction could be shown by testing for elevated mortality risk following the production of offspring. For example, early studies suggested that producing offspring is correlated with reduced lifespan among parents of both sexes (28, 29), though in general the strongest evidence is for mothers. However, several studies have failed to demonstrate negative associations between reproduction and lifespan (123, 124), and the evidence that greater reproductive effort promotes faster ageing through oxidative damage is inconsistent (26). We suggest that a wider range of metabolic traits relative to fitness merit consideration.

Reproduction is a challenging period for maternal metabolism, temporarily depleting the mother of energy, micronutrients, and mineral. For example, higher parity, short inter-birth interval, and earlier age at first birth were associated with reduced bone quality among Tsimané forager-farmer women after adjusting for potential confounders (125). These findings are especially relevant to our hypothesis, as bone mineral density can potentially be examined in the archaeological skeletal record. However, studies from high-income countries indicate that the net loss of bone during lactation may be resolved after weaning (126). Moreover, other studies of the Tsimané found that despite their high fertility rates, markers of cardio-metabolic disease are amongst the lowest reported in human populations (127, 128). The costs of reproduction may therefore be both “condition dependent,” i.e., varying in association with broader ecological conditions, and also outcome-dependent, i.e., varying across different markers of maintenance (26). In addition, they may also be shaped by experience in early life. For example, the effect of activity level on reproductive function in rural Polish women was found to be mediated by size at birth (129).

Parent-offspring conflict theory assumes that offspring are selected to demand more resources than their parents are selected to provide (130). During pregnancy, this results in a “metabolic battle” over maternal circulating nutrients. The fetus and placenta (which share a common genotype) secrete hormones that increase maternal glucose levels and blood pressure, which act to force more nutrients across the placenta. The mother responds by counter-effects, reducing the pool of nutrients (131). The metabolic strain of pregnancy makes mothers vulnerable to conditions that impair maintenance, such as gestational hypertension and diabetes. Whilst these metabolic conditions are strongly associated with obesity in contemporary populations, there are indications that they also affected past populations, perhaps through the adoption of diets that exposed metabolism to unprecedented levels of refined carbohydrate (132). Any metabolic costs of particular diets to the mother are expected to have been exacerbated by the effects of maternal-offspring conflict.

For cardio-metabolic outcomes, therefore, reproduction appears to increase the risk of chronic diseases in women, indicating that it imposes costs on homeostasis. However, these costs may to some extent be mitigated by breast-feeding (133), moreover reproduction is protective against diseases associated with excess fuel availability, in particular cancers (134). Therefore, trade-offs between reproduction and maintenance vary in association with the underling metabolic pathways to disease. Intriguingly, both short and long inter-birth intervals have been associated with elevated maternal mortality risk (135).

Some costs of reproduction can potentially be offset by greater kin support, as expressed in the concepts of cooperative breeding (136) and pooled energy budgets (118). In this context, sedentary farmers might be able to draw on a larger pool of relatives than foragers, while also benefitting from new cereal-based weaning foods (137) that could promote such kin-cooperation. Conversely, the costs of reproduction could also be elevated by shorter inter-birth intervals, hence markers of health and longevity must be assessed to test whether the transition to agriculture was beneficial or detrimental to “maintenance” in women.

### Maintenance-Defence (M-D)

Defence typically requires that baseline homeostatic processes be curtailed in favour of more aggressive metabolic activities, that either protect the body from external threats (predators), supply damaged tissue with resources, or neutralise pathogens and parasites.

The generic costs of immunity have been elegantly revealed through studies of non-human animals, that for ethical reasons are not appropriate in humans. For example, a study of bumblebees showed that, after imposing starvation to ensure limited energy availability, simply activating the bee's immune system in the absence of actual exposure to pathogens reduced survival of the bees by 50–70% (138). Immune function can therefore be regarded as a high-benefit, high-cost trait, that is potentially life-saving but metabolically expensive to run (139). Similarly, many experimental studies have shown that injecting animals with foreign antibodies generates an elevation in metabolic rate, which clearly reduces the availability of energy to other functions (140, 141).

In young men, observational studies showed that even mild respiratory infection increases resting metabolic rate (142). In children, likewise, each degree of temperature rise associated with fever increases metabolic rate by ~11% (143). A recent study of Shuar forager-horticulturalist children of Amazonian Ecuador found resting energy expenditure to be increased by ~20% relative to children from industrialized settings, due to persistent immune activation (144). At the level of cellular metabolism, injury or infection elicits a state of inflammation, disrupting homeostatic processes such as the maintenance of core body temperature, appetite and sleep patterns (139). These responses impair components of cellular homeostasis such as DNA repair and telomere maintenance (145, 146).

The costs of defence relate not only to immune function itself. Many pathogens may not necessarily threaten survival, but nonetheless rely on their hosts for nutrition, shelter, warmth, and a “home base” for reproduction. Until cleared from the body, all their metabolic requirements are necessarily met by the host organism (147). Given the high costs of prolonged immune response, the optimal trade-off may be to tolerate some parasites or pathogens (148, 149). The lower the level of energy supply, the higher may be the resulting tolerated pathogen burden. This issue is particularly relevant to early agricultural communities, as they experienced unprecedented exposure to pathogens and parasites compared to ancestral foragers.

From a behavioural perspective, the stress response plays a key role in enabling escape from predators, but again at a cost to normal homeostatic function (150, 151). The hormone cortisol plays a key role in allocating energy between different physiological systems. High cortisol levels maintain alertness and the capacity to respond to stresses, but at a cost to cardio-metabolic health (152–154).

The study of Mayan children discussed above showed that children with higher levels of physical activity not only demonstrated poorer growth, but also had reduced subcutaneous adiposity, indicating that working harder on subsistence tasks reduced allocation to immune function (122). In extreme conditions, however, physical activity could itself be considered an investment in defence. One such example comprises fleeing from predators, however farmers may also need to work especially hard in some seasons to reduce the risk of famine (155), or protect crops from insect pest invasions. In contrast to moderate activity levels, intense levels can cause weight loss (156, 157), and can result in the net production of free radicals, causing oxidative damage (158).

Beyond direct energetic costs, greater investment in immunity may also compromise other nutrient-dependent forms of maintenance. For example, among Tsimané forager-horticulturalists in Bolivia, markers of elevated immune activation were associated with estimates of lower trabecular bone density, a risk factor for fragility fractures at older age (159). Although exposure to pathogens in early life may also contribute, the markers of immune activation in this study were measured during adult life, and indicate continued deficits in bone maintenance generated by the burden of infections.

### Growth-Reproduction (G-R)

At the simplest level, reproduction broadly occurs only when growth has ceased, meaning that the starkest trade-offs are driven by a time-shift in allocating energy between these functions. However, considered in more detail, there are more subtle trade-offs between these functions.

First, there may be a genetic basis to a trade-off between maturation rate and adult size. Both stature and age at menarche demonstrate heritability (see Table 1), and short stature has been correlated with earlier menarche (160, 161). This suggests that some populations might have adapted to high-risk environments by shifting the G-R trade-off systematically in favour of earlier reproduction (33). Within populations, genetic variability in these traits indicates a range of variability in this trade-off (162). However, the same trade-offs can also emerge through plastic mechanisms.

First, early reproduction appears to curtail maternal physical growth. Several studies have shown that adolescent childbearing is associated with a reduced rate of linear growth, indicating that the energy costs of reproduction reduce the allocation of energy to maternal growth (163). Second, several studies have shown a trade-off between weight gain and height gain. For example, age at menarche is positively correlated with adult height (161, 164), but negatively correlated with adiposity through adult life (165). This indicates that the developmental pathway to earlier reproduction favours the allocation of energy to somatic stores, at a cost to linear growth. Whereas stature and lean mass are markers of growth, gluteo-femoral adipose tissue can be considered an investment by females in reproduction, providing energy stores to fund lactation (59, 166).

Catch-up growth allows the body to respond to early under-nutrition, should more resources become available. However, studies show that rapid catch-up growth may promote adiposity over linear growth. For example, studies of Indian girls who were adopted by Swedish families in early life showed that in the improved nutritional environment, they underwent very early puberty and remained short as adults (103, 167). Again, this highlights the diversion of resources from growth and maintenance toward earlier reproduction.

### Growth-Defence (G-D)

Numerous studies in children show that infections reduce linear growth rate, examples including helicobacter pylori infection and diarrhoea (105, 106, 168). Among Shuar forager-horticulturalist children in Amazonian Ecuador, even mildly elevated immune activity reduced growth rate by half (106). In the reverse direction, childhood immunisation programmes are beneficial for child growth, through reducing the allocation of energy to fighting infections (169). Aside from linear growth, infections can also reduce tissue masses. In acute illness, for example, in the absence of adequate dietary supply, lean tissue may be broken down to release acute-phase proteins. Similarly, populations occupying environments with higher infectious burdens show lower levels of truncal subcutaneous fat (170), a depot closely associated with immune function (60, 171).

From an inter-generational perspective, maternal infections during pregnancy also reduce the energy available for fetal growth (172). Numerous studies have linked maternal pregnancy infection with lower birth weight (173, 174), and these associations persist into post-natal life. For example, infants exposed to maternal HIV, but themselves uninfected, show poor growth during early infancy, the period of exclusive breastfeeding (175). Placental malaria likewise constrains infant catch-up growth (176).

These trade-offs may generate correlations between the burden of infectious disease encountered in early life, and subsequent adult height. Many studies have assessed childhood infection burden through the proxy of infant mortality rate, on the assumption that higher infant mortality indicates exposure to a higher disease load amongst those who survived. Over the twentieth century, declines in infant mortality rate within countries correlate strongly with increases in adult stature 20 years later (89). While these studies are observational and cannot prove causation, they support the hypothesis that linear growth benefits from less energy being allocated to immune function, consistent with the mechanistic studies reviewed above.

When dietary quality improves in the absence of increased infection burden, more energy can be allocated to growth. For example, among moderately malnourished young children in Burkina Faso, providing high-energy ready-to-use therapeutic foods along with medical care resulted in 93% of weight gain comprising lean tissue, indicating prioritised allocation of energy to growth (177, 178).

### Reproduction-Defence (R-D)

Immediate trade-offs between reproduction and defence are illustrated by the greater susceptibility to infections among women during pregnancy and lactation. For example, the energy demands of lactation make mothers more susceptible to malaria infection during the early post-partum period (104).

As with growth, greater exposure to infections in early life can slow the rate of maturation and hence potentially delay reproduction. For example, Ellison reviewed data on infant mortality rate in the 1940s, and mean age at menarche in the 1960s−1970s, in populations from low- and middle-income countries (179). Among populations where mortality was generically low, there was no association between infant mortality and age at menarche. Above a certain threshold of infant mortality, however, there was a dose-response linear correlation between the two parameters. This implies that in populations suffering a high disease burden, expending more energy fighting infections slows the rate of maturation.

However, growth-defence trade-offs can also lead to *earlier* menarche, which may in turn result in shorter adult height. As discussed above, maternal infections during pregnancy may reduce fetal growth, propagating to shorter adult height of the offspring. Catch-up growth may exacerbate this effect, by accelerating pubertal development but thereby shortening the duration of growth (164). Both of these R-D trade-offs could have operated in populations undergoing the transition to agriculture.

Defence may also relate to psychosocial factors associated with the stress response. Activating the “flight-or-fight” response reduces energy availability for other functions. Studies have associated maternal stress during pregnancy with lower birth weight (180). A recent randomised trial showed that reducing anxiety among healthy first-time mothers was associated with increased breast-milk transfer, and with greater weight gain in the infant (109).

### Composite Trade-Offs and Inter-Generational Effects

So far, we have considered evidence for binary trade-offs between life history functions. Few studies have considered how ecological factors shape “bundles” of trade-offs more comprehensively, however we review several examples highlighting the relevance of life history trade-offs for understanding the potential consequences of variability in ecological conditions. None of these studies explicitly examines the consequences of change in human subsistence mode, but each shows how variability in ecological conditions is associated not simply with variability in a specific trait, but rather in composite life history strategies that respond through genetic change or reaction norms to maximise fitness. Our emphasis here is that coherent trade-offs, in response to particular selective pressures, are expected to result in multiple traits clustering within individual organisms.

One such example has been observed in non-human animals, and relates to the emergence of distinct “animal personalities.” This has been attributed to the action of selection on traits that coordinate risk-taking behaviour (181). A similar scenario may relate to suites of life history traits in human populations.

A second example goes beyond the traditional focus on energy allocation, to consider dietary macronutrient composition. The framework of “nutritional geometry” assumes that animals satisfy competing appetites for different macronutrients in ways that maximise fitness (182). In Drosophila, diets that maximised longevity had different composition to those that maximised fecundity. When offered a choice of complementary foods, flies regulated their food intake to maximize lifetime egg production (183). Similar experimental work on mice has likewise shown that dietary macronutrient composition effects both health and longevity (184). Changes in the diet therefore appear to drive composite changes in life-history trade-offs in non-human animals.

A third example comprises a study of 22 small-scale human societies by Walker et al. (33). This study showed that variation in both the supply of energy, and mortality risk, is associated with varying patterns of growth, indicating that environmental conditions drive trade-offs across populations. The authors identified one subset of societies, occupying more favourable conditions, which demonstrated faster growth and earlier puberty. These populations attained adulthood faster because of greater energy availability, proxied by larger adult size. However, the authors also identified another subset of populations that experienced low sub-adult survival rates. In this subset, earlier maturation and reproduction is again favoured to counter mortality risk, but at a cost to adult body size. The authors concluded that both genetic adaptation and life-course plasticity might contribute to these contrasting strategies. Individual studies have elucidated in more detail several relevant trade-offs. For example, among Pume foragers in Venezuela, early female reproduction is favoured by a rapid growth spurt prior to the adolescent onset of reproduction, and the provision of food by kin (energy-pooling) to meet the metabolic costs of this fast life history strategy, which collectively maximises female fitness (185–187).

**Figure 3:**
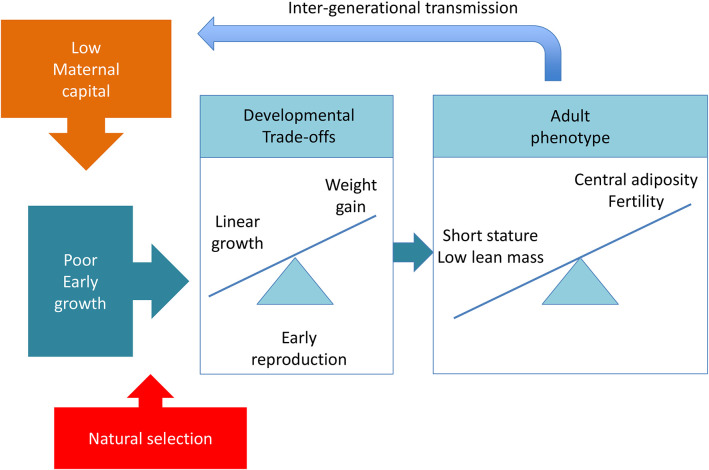
Summary of findings from the Pelotas 1993 birth cohort study, where low maternal capital was associated with developmental trade-offs in the daughter between linear growth and weight gain. At 18 years, daughters showed preferential energy allocation to reproduction and defence, at a cost to growth and maintenance. Based on data from Wells et al. (188).

A fourth example illustrates how these trade-offs may emerge through the life course, in response to variable investment in early life. In a longitudinal cohort study from Brazil (188), lower levels of maternal investment were associated with developmental trade-offs that favoured immediate survival and early reproduction at a cost to growth and maintenance (Figure 3). Maternal capital was assessed by scoring “penalties” in each of maternal height, nutritional status, family income, and education level. A composite score of these penalties enabled mothers to be ranked in terms of overall capital level, assumed to equate to variable capacity for maternal investment.

Lower-capital mothers produced daughters with smaller size at birth, who continued to show poor linear growth during infancy. Compared to daughters of high-capital mothers, the low capital daughters did not experience earlier menarche, but nevertheless were more likely to have produced offspring by 18 years, while being both shorter and more centrally adipose in early adulthood. This study highlights a life-course developmental trajectory of growth being curtailed from fetal life onwards, and energy instead being allocated to body fat to fund reproduction (peripheral fat) and immune function (central fat). Overall, low maternal investment drove trade-offs that promoted reproduction and defence at the expense of markers of maintenance and growth.

**Figure 4:**
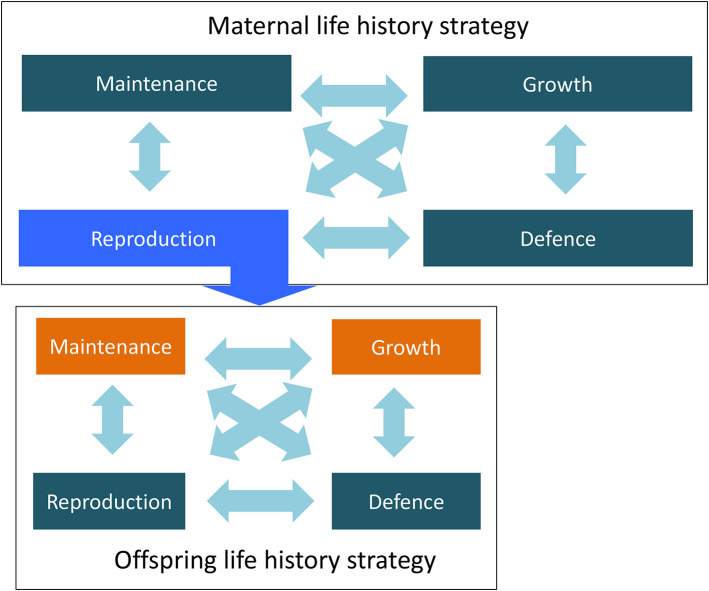
Life history trade-offs across two generations, showing how the relative allocation of energy by the mother to reproduction shapes the energy available for allocation between all four functions in early life in the offspring.

This study illustrates how reproduction brings the life history strategies of two generations together. The mother's allocation of energy to reproduction is shaped by her own life history trade-offs, while the magnitude and developmental timing of this investment shapes the cumulative emergence of trade-offs in the offspring (Figure 4). In that sense, the daughters' trade-offs are responses to trade-offs occurring during maternal development.

Having demonstrated comprehensive evidence in support of binary, composite and inter-generational trade-offs in contemporary human populations, we now turn to the origins of agriculture to consider whether there is also evidence for such trade-offs in association with major changes in human diets and living conditions.

## The Origins of Agriculture

It is now generally recognized that the transition to agriculture involved a long-term co-evolutionary relationship that increased the population size and density of both humans and their domesticated plant and animal species over thousands of years. This process, where it occurred, involved the replacement of foraged and hunted foods with domesticated varieties and animal by-products, and involved the gradual selection for larger grain size indices representing greater agricultural productivity (189). However, it is also important to note that a proportion of human populations never adopted any form of agriculture, others did so only transiently, and still others practised mixed foraging and farming (190, 191). Where agriculture did emerge, it did so in a wide variety of ways and on different timescales, and can therefore be assumed to have impacted human biology in heterogeneous manner. Wherever it occurred, the association between niche construction and human biology is likely to have involved positive feedback, so that farming stimulated new life history trade-offs that may then have shaped the subsequent trajectory of agricultural development.

Domestication involved “a continuum of human, plant, and animal relationships … and was driven by a mix of ecological, biological, and human cultural factors” (6). Its timing varied substantially across different geographical regions, and whereas in some (e.g., the New World) crop domestication preceded that of animals by several millennia, in others (e.g., Africa, Arabia, India) the converse occurred (6). The role of active human selection for specific traits also varied, and some traits that were beneficial for humans likely emerged as a by-product of cultivation/husbandry practices (6). Given this heterogeneity, we should expect human life history traits to have shifted, by genetic or plastic mechanisms, whenever the changes to the socio-ecological niche were of sufficient magnitude to favour such responses. Which periods generated the greatest selective pressures, opportunities, or stresses, and hence drove the most marked life history shifts, is an important topic for further work.

With richer and more stable resources and larger social groups aggregating at specific settlements, storage of food surpluses, new forms of cooperative behaviour, and the exploitation of renewable dairy animal by-products, the transition to agriculture dramatically shifted the energetic ecology of the human dietary niche. The human gut is small in size with a limited transit time, thus constraining the volume of food that can be ingested and, through digestion, converted to metabolisable energy. By consuming foods that are energy-rich and extra-somatically processed (e.g., ground grain/carbohydrate and milk), dietary energy supply can be increased despite our biological constraints.

However, beyond dietary shifts *per se*, any observed changes in human biology that occurred in association with the transition to agriculture should be considered in the context of changes in the entire ecosystem. Human life history transformations occurred alongside similar changes in a variety of the organisms that were farmed. Through the process of domestication, humans actively or passively selected for and against many of the traits that represent life history adaptations of crop and animal species.

For example, human activities changed the morphology of plants in favour of increased grain sizes and non-shattering spikelet scars of wheat, barley, and rice (189). This had the effect of producing larger, more energy-rich grains that were less likely to be lost in harvesting, but often required further processing before consumption. Moreover, by selecting against components of plant and animal “defence,” humans had to invest more time and effort in defending their new resources against the pathogens and predators that target these species. Over thousands of years, early farmers were therefore drawn into a new “labour trap,” and exposed to new stresses associated with enhanced seasonality of the food supply (192).

**Figure 5:**
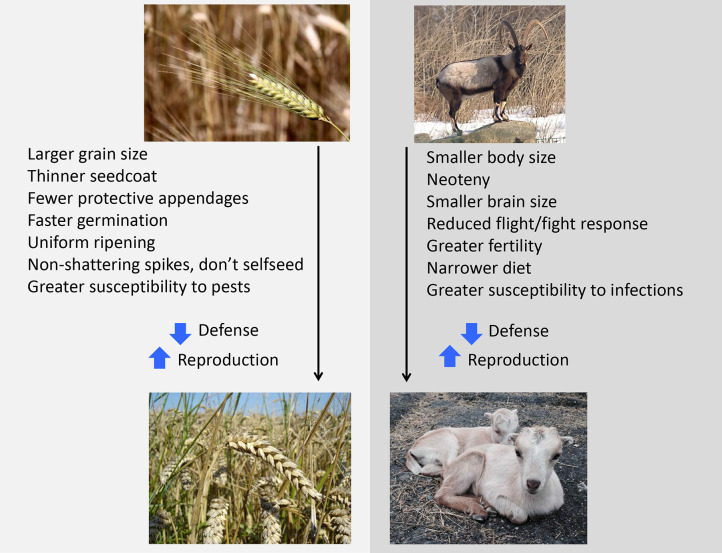
Trade-offs between traits in crop and domesticated animal species, reflecting artificial selection by humans during the early agricultural period (192, 193). Photo credits (**Top left**) LepoRello (https://commons.wikimedia.org/wiki/File:Triticum_boeoticum_Bajuwarenhof_Kirchheim_2012-08-05.jpg), “Triticum boeoticum Bajuwarenhof Kirchheim 2012-08-05,” https://creativecommons.org/licenses/by-sa/3.0/legalcode (**Botttom left**) User:Bluemoose (https://commons.wikimedia.org/wiki/File:Wheat_close-up.JPG), “Wheat close-up,” https://creativecommons.org/licenses/by-sa/3.0/legalcode (**Top right**) F. Spangenberg (Der Irbis, own photo) (https://commons.wikimedia.org/wiki/File:Bezoarziege.jpg), “Bezoarziege,” https://creativecommons.org/licenses/by-sa/3.0/legalcode (**Bottom right**) Cleur Monie (https://commons.wikimedia.org/wiki/File:Lamancha_mix_goat_kids.jpg), https://creativecommons.org/licenses/by-sa/4.0/legalcode.

In these respects, domestic plant and animal species showed their own life history shifts whereby investment in defence was suppressed, while investment in the traits that from a human perspective drive agricultural yield increased (Figure 5). In crops, this is reflected by larger grain size, whereas the size of animals often decreased initially (193) while their fertility increased (192). In each case, these trends indicate greater investment in reproduction, and hence greater potential harvests for humans. This evidence indicates that humans may have changed through similar correlated shifts in life history trade-offs, allowing adaptation to the new agricultural niches.

Foragers diversify their efforts across multiple food webs, and are protected against shocks in any one of them (194). In contrast, farmers increasingly invest in a single food web, and become more susceptible to any ecological stress that reduces its productivity (192). Agricultural settlements are often near natural watercourses, which allowed for the development and intensification of irrigation to maintain crop yields, which created more larval habitats for vector-borne diseases (195). These concentrated communities may then have seen a further intensification of the infectious burden, radically transforming the risks of morbidity and mortality.

### Composite Stress Imposed by Agriculture

The adoption of agriculture transformed the entire human subsistence niche, changing both the human diet and many other aspects of the local ecology, which we argue may have led to a cascade of coordinated life history trade-offs. However, these changes must have played out in varying ways according to the historical period, the local ecology, and the type of agriculture that developed. As all of these factors would have been under the influence of longer-term climatic trends, the selective pressures must therefore have varied accordingly. We briefly summarise some of the key stresses and some of the trends that might have shaped them.

Compared to forager diets, those of early farmers tended to incorporate higher levels of carbohydrate from grains, but lower levels of fibre, micronutrients, and protein (9, 20, 196). These changes would have altered the macronutrient substrates available for metabolic processing, with implications for life history trade-offs as highlighted above regarding experimental work on non-human species (183, 184). In humans, for example, low levels of dietary protein are associated with slower childhood growth (197, 198) and with higher levels of fat storage (182, 199). In this context, the implications of dairying are of especial interest. Following the emergence of a specialised dairying economy in the European Steppe by 7000BP, single nucleotide polymorphisms (SNPs) associated with lactase persistence appear to have evolved by ~5600BP (200). In particular, the adoption and spread of intensive dairying may have buffered the difficulty of agricultural subsistence in Northern Europe and led to the modern north-south gradient of body size in Europe, an interpretation supported by the detection of selection for reduced height in the Iberian Neolithic but increased height in the Neolithic populations of the steppe (201).

Agriculture also exposed human populations to greater seasonality in food supply, exacerbated by the risk of famine through harvest failure. Other seasonal stresses that could dramatically reduce annual yields include floods, or spikes in agricultural pests.

A second key stress experienced by growing sedentary populations comprised exposure to a range of pathogens (195), driven by several related factors. First, higher population densities inherently favoured greater opportunities for infection. This scenario was then exacerbated by greater exposure to pathogens associated with human/animal faeces and contaminated water sources, and by the proximity to domesticated animals, some of which transmitted novel diseases to humans. Indeed, the longer the history of domestication of a species, the more common infectious diseases they share with human populations (202), indicating a long history of exposure to zoonotic disease following domestication. However, although early farming populations are widely assumed to have acquired an elevated burden of pathogens from their newly domesticated animals, emerging evidence suggests they may also have passed pathogens adapted to humans back to their stock animals, one example being the transfer of salmonella to pigs (203). Human populations also became susceptible to new “crowd” infections that, since they infect people only briefly before they recover or die, require a relatively large population size for their persistence (204), and against which foragers had been protected through their nomadic lifestyle and small population size. This enhanced overall disease load had two key effects on life history strategy—first, it increased the energy demand for immune function, and second it increased extrinsic mortality risk, which would then favour earlier reproduction (either achieved through maturing earlier, or through ceasing growth at smaller size). Each of these effects would inherently reduce the energy available for growth and maintenance.

Over the longer-term, climate change altered seasonal patterns and extended the dry season, leading to agricultural intensification and the adoption of practices such as mass irrigation (205). These more concentrated communities may then have experienced greater susceptibility to the stresses highlighted above.

Finally, there is growing evidence that the ecological stresses associated with the transition to agriculture may have intensified under the influence of early states, and that their political institutions may have influenced the crops grown, the diet consumed, the extent of crop irrigation, and the risk of disease and subsistence crises (192). Furthermore, states presupposed growing levels of social inequality, and state control over resources.

Since farming can increase dietary energy supply relative to foraging, one could question whether the transition to agriculture must inevitably have driven life history trade-offs. Could not the additional energy costs of immune function have been met simply by consuming more calories? Alternatively, farmers could have demonstrated lower physical activity levels, thus reducing their energy demands, for example by benefitting from new “economies of cooperation” that are less amenable to exploitation by individual foragers (9). However, a review of energy expenditure in contemporary subsistence farmers suggest that levels of energy expenditure are moderate to high (206), while a study of Hadza foragers found that their energy expenditures were lower than expected (207), despite high levels of physical activity. Food production generates new demands for “food processing,” meaning that farmers may have to work harder to produce the same amount of dietary energy as foragers. Contemporary subsistence farmers also demonstrate prevalences of child malnutrition that are amongst the highest of all human populations (208), indicating that the composite stresses of food insecurity and infections is detrimental to growth. This is an important point, as many ecological stresses relevant to the transition to agriculture may have acted most strongly during early development, rather than during adult life. Finally, trade-offs could have occurred in response to changes in dietary macronutrient composition, as well as in the overall energy budget. For all of these reasons, we therefore consider that phenotypic shifts mediated by trade-offs were likely inevitable in early farmers. The mechanisms could have allowed phenotypic responses favouring growth and maintenance during better ecological conditions, and the reverse pattern during more stressful periods.

**Figure 6:**
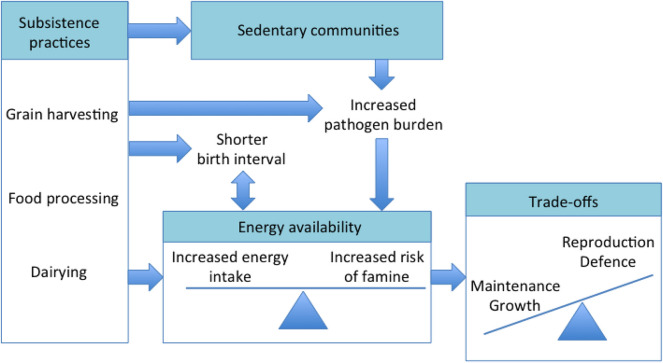
Summary of how the combination of changes in subsistence practices may have increased energy availability, but also changed the ecological stresses in early agricultural populations. These composite changes may have elicited life history trade-offs favouring reproduction and defence, over maintenance and growth, as described in detail in the text.

Overall, we can assume the emergence of agriculture changed the human diet while provoking profound life history trade-offs that increased the allocation of energy to reproduction and defence, at a cost to growth and maintenance, as illustrated in Figure 6. We now review evidence in favour of each of these trends.

### Reduced Allocation to Growth

There is relatively consistent evidence for a decline in adult body sizes associated with the transition to agriculture (209–214). A recent systematic review found evidence of declining stature in 14 different analyses among populations from Europe, Africa, the Middle East, Asia, Central and South America, and North America (215). While the trend toward decreasing stature is commonly associated with the transition to agriculture, there is some evidence for temporal and regional variation. In some cases the initial transition to agriculture was associated with an early small increment in stature, followed by later, long-term systematic decline (9); or more subtle patterns of decline that varied between men and women (216). In other regions stature remained relatively consistent across the transition or even increased (217). Some of the cases where more complex patterns are observed involved the transition to wet-rice agriculture that may have had different energetic consequences, both in terms of the high energetic demand of paddy field farming, but also higher yields and lower amylose content (218, 219), while others may have reflected broader socio-economic changes in the Holocene.

More recent studies have analysed long diachronic samples. One such study shows that within the central European steppe, there was a significant decline in stature between the Mesolithic and Neolithic (220) that persisted among both men and women through the bronze and iron ages before a recovery in the Medieval period. Similarly, height declined sharply in association with the adoption of agriculture in India, and has remained low subsequently (221). Another recent study reported a similar decline in stature among the earliest farmers in the Nile Valley, followed by a subsequent increase in stature with the rise of the Egyptian Empire (222), trends that are matched by evidence for periods of childhood stress (223).

These bulk of studies typically document a decline in stature that is either immediately associated with the agricultural transition or occurs with agricultural intensification. This trend appears to persist in many contexts for thousands of years before an eventual increase. In each case, the initial size reduction demonstrates decreased energetic investment in somatic growth, which suggests a shift in life history strategy following the transition to domesticated plant and animal resources. Overall, therefore, the available evidence suggests that in most regions the allocation of energy to somatic growth initially declined in association with the transition to agriculture, but was followed by increases associated with subsequent shifts in energetic ecology.

Since height in many populations has recently increased, it is not clear whether the declines associated with adopting agriculture involved genetic adaptation, although there is some evidence for a general correspondence between stature estimates and polygenic risk scores for genes associated with stature (224). Intriguing evidence comes from inter-ethnic studies of birth weight, where the ethnicity of each parent can be considered separately by comparing offspring with parents of contrasting ethnicity. In this study, infants with European mother and south Asian father weighed less than infants with two European parents, suggesting that in the Indian population, genes expressing the paternal growth drive may have been selected to demand a lower nutritional transfer from the mother during fetal life (225). This may relate to the challenges of developing agriculture in an environment with high ecological volatility associated with the monsoon. Further studies are needed to test this hypothesis more robustly.

### Increased Allocation to Reproduction

It has long been considered that there is a causal relationship between subsistence strategies, as the basis for the mode of production, and demographic change, with agricultural subsistence directly leading to more permanent settlement and hence the demographic expansion of populations (16, 226). However, prehistoric demography is challenging to interpret, as it is dependent on proxy data. Many early estimates of exponential growth in human populations were based on evidence from rapid increases in settlement sizes, but recent use of radiocarbon dates as proxies for demography highlight more subtle fluctuations of population in some regions throughout the Holocene (227).

The strongest evidence for population growth in the Holocene comes from direct analysis of human remains and modern human genetic diversity. In the most systematic study of Neolithic demography, for example, Bocquet-Appel compared palaeodemographic data from 200 cemeteries (228). The results suggest there was a relatively abrupt increase in fertility following the transition to agriculture in the Northern Hemisphere. In the Levant, this is estimated to represent an increase in total fertility from 4.5 to 10 throughout the reproductive lifespan (228). The notions that fertility increased and inter-birth intervals decreased are supported by ethnographic studies of demography among recent or contemporary foragers and transitional-farmers (229), and by comparisons across subsistence mode that control for phylogenetic relationships (230).

Recent evidence for an agricultural demographic transition also comes from genetic estimates of population sizes. For example, Gignoux and colleagues investigated mitochondrial DNA diversity and revealed strong evidence for demographic expansions in the past 10,000 years in Europe, south east Asia, and sub-Saharan Africa (231). In all cases, coalescence times linked these demographic expansions closely with the adoption of agricultural subsistence.

Evidence regarding the effect of the transition to agriculture on mortality patterns is less consistent. Comparing palaeodemographic life tables of hunter-gatherers, horticulturalists, and agriculturalists, mean life expectancy was 21.6, 21.2, and 24.9 years, respectively, with none of the differences being statistically different (232). However, we should also note that mortality rates before and after the transition to agriculture might not necessarily be the same as those during the transition, and there are many uncertainties that are difficult to resolve when estimating past mortality rates (232). Moreover, the implications of transitioning to agriculture may not necessarily have been equal for the two sexes. In a study of age at death in the Levant, for example, life expectancy of Neolithic populations appeared to be slightly greater than that of the earlier Natufian hunter-gatherers. However, relative to males, female longevity appeared to decline, suggesting an elevated burden of maternal mortality in the Neolithic (233).

Importantly, however, our conceptual framework is relatively robust to this uncertainty. As discussed above (Box 1), we do not need to assume a simple linear correlation between health and lifespan. Rather, rising rates of markers of disease in bone among early agricultural populations could simply reflect that people typically lived in poorer states of health. Since early farmers do not appear to have lived significantly longer than their hunter-gatherer predecessors, elevated frequencies of pathological indicators are unlikely to be an artefact of a new reservoir of older individuals, in whom such deterioration would be expected regardless of their subsistence niche, but rather indicate higher levels of morbidity throughout a similar lifespan.

Collectively, therefore, there is strong evidence for a major demographic shift associated with the origins of agriculture, driven primarily by rising fertility rates. While it is expected that higher resolution data will reveal subtle and minor regional variations to this trend that are dependent on local circumstances, there is no doubt that the transition to agriculture was accompanied by a significant demographic shift that stimulated the population growth of the last 10,000 years.

### Increased Allocation to Defence

There is a significant body of evidence that many of the most significant infectious diseases that afflict human societies originated in other species, were propagated by the process of domestication, or found enhanced environments for vector-borne transmission following the transition to agriculture (234, 235). There is also a demonstrable link between agricultural land use and infectious disease risk today (236).

The impact of these diseases on human populations is demonstrated by genetic evidence, which suggests that pathogens have been the main selective pressure in recent human populations (237, 238). Palaeopathological evidence from prehistoric archaeological sites is consistent with the hypothesis of increased exposure to pathogens among early farmers. An early, and now classic, synthesis of research in this area identified widespread increases in markers of disease associated with the transition to agriculture in different regions (20). While some of the assumptions of this interpretation have been challenged (239), the general observations have been repeated in other regions and very large datasets (240) suggesting that the relationship between the agricultural transition and exposure to infectious disease is widespread and consistent.

More recent comparisons of hunter-gatherer and Neolithic skeletons spanning the earliest origins of agriculture in the Levant have demonstrated an increase in pathological conditions causing inflammatory lesions among the earliest farmers, and this has been interpreted as evidence for heightened immune function in response to pathogen exposure (241). The most significant recent review of palaeopathological evidence for infectious disease following the transition to agriculture demonstrates increases in the prevalence of four infectious diseases that are slow to progress and leave signatures on the skeleton: treponematosis, tuberculosis, dental caries, and periodontal disease (242). These infectious diseases generally represent chronic conditions that cause consistent, long-term effects on human health, and therefore represent markers of elevated morbidity rather than overt mortality risk and shorter lifespans (as discussed above). Their slow progression in part explains the fact that they are manifest in skeletal lesions, as the skeleton is slow to remodel and only reflects conditions over a long period of time. Such diseases would have necessitated heightened and sustained immune response, which as discussed above would be energetically costly.

The long-term energetic costs of pathogen response could be exacerbated by the evolution of pathogens themselves. Pathogens may become more or less virulent through time, depending on mechanisms of transmission, morbidity, mortality, and the frequency of epidemic waves. If an infection immunizes those who survive, and returns at a relatively short interval of 5–10 years, then it will automatically become a childhood disease. One consequence of this, observed both in mathematical models and in recent demographic datasets, is that adult life expectancy may increase even as life expectancy at birth declines (243). Using average lifespan as a marker of investment in defence is therefore of limited value, and markers of skeletal health in different age groups merit more attention. This underscores the importance of demography to our interpretation of palaeopathological data in the archaeological record (244).

An increased parasite burden would also place energetic demands on the host. Recent evidence demonstrates for example the presence of whipworm at the early farming community of Çatalhöyük in modern Turkey (245). In sum, the prehistoric impact of pathogens on human populations seems clear, both in the increased burden of infectious disease, and the energetic consequences of the immune response.

### Reduced Allocation to Maintenance

In contrast to the three life history functions considered above, it is more challenging to interpret changes in energy allocation to maintenance in the past, as the only remaining biological tissues are typically bone and teeth. One possible approach is to consider markers of bone maintenance. Recent evidence documents a general decline in the mechanical competence of the skeleton associated with the transition to agriculture, both in cortical (222, 246) and trabecular (247) bone. While this is perhaps best interpreted in relation to decreasing mechanical loading of the skeleton and dietary shifts, it also reflects a decreased investment in skeletal tissue remodelling throughout the adult lifespan, and thus decreased investment in skeletal maintenance.

While it is difficult to identify other specific markers of cell maintenance in the past, we can draw on physiological studies in living humans to interpret archaeological evidence. One measure of maintenance is antioxidant capacity, which fights the accumulation of free-radicals that are associated with multiple diseases. While antioxidant profiles have not been sufficiently compared between hunter-gatherers and agricultural populations, there is evidence that more homogenized diets with lower diversity of plant foods lead to lower antioxidant levels (248), and that antioxidant levels are inversely proportionate to cancers (249). Likewise, higher antioxidant levels appear to prevent low-density lipoprotein oxidation, which delays the onset of atherogenesis and progression of atherosclerosis (250). This evidence is suggestive of an association between dietary shifts and a decrease in measures of somatic maintenance.

One line of evidence that can illuminate this issue comes from the analysis of mummified human remains. A recent study of 137 mummified humans from recent ancient populations from Egypt and Peru, and recent ancestral populations in southwest America and the Aleutian Islands, demonstrated the presence of atherosclerosis in 34% of all individuals, with a prevalence ranging from 25 to 60% within populations (251). This study found high frequencies of atherosclerosis among several agricultural populations. While the Aleutian Islanders included in this study practiced a hunter-gatherer subsistence strategy, their diet was also very high in animal protein and fat as is typical of arctic foragers. At this stage, there is no similar prehistoric evidence from terrestrial or marine foragers at lower latitudes, however living Tsimané forager-horticulturalists from Bolivia show low levels of coronary atherosclerosis (128). How the transition to agriculture affected cardiovascular health therefore remains unclear, and might demonstrate heterogeneous effects.

More broadly, further work is required to clarify trends in the allocation of energy to maintenance. However, under the logic of the capacity-load model (252), reduced linear growth can also be considered a marker of depletion of maintenance in the long-term. Growth is most sensitive to insults in early life, and this is a key period for the development of the metabolic capacity for homeostasis (252). During development, growth is associated with organ size (253), and in adulthood, shorter adults have smaller organs and poorer capacity for metabolic homeostasis (117, 254). Thus, the declines in growth described above provide indirect evidence for reduced energy allocation to maintenance.

Of relevance here, the allocation of energy to maintenance also involved new forms of pooled energy budgets (118), where both adults and children could undertake specific subsistence tasks. On the one hand, parental subsistence activities may have increased the supply of energy to meet the maintenance costs of children, for example by developing food stores that could feed entire households during “hungry seasons.” On the other hand, farming also provided new opportunities for children to contribute to subsistence effort, for example by shepherding domesticated animals, or by gleaning crops at harvest time. The energetic consequences of variation in habitual activity, as a component of both intra-and inter-individual life-history trade-offs, is an area that requires further research.

In summary, the preponderance of evidence suggests that there were general and coordinated life history shifts associated with the transition to agriculture, supporting the overall trends illustrated in Figure 6. Agricultural subsistence generated more energetically-rich food through the processing of grain and through secondary animal by-products like milk. The energetic and mechanical properties of this diet, in combination with the storage of surpluses, ensured the perpetual availability of weaning foods, and led to shorter inter-birth intervals. Agricultural communities were also typically sedentary which, in combination with living in close proximity to domestic animals, increased the pathogen burden. The general features of agricultural societies led to increased energetic availability in general, but also an increased risk of famine, and overall characteristics of the environment that lead to life-history trade-offs. From the review above, we note that the transition to agriculture appears to be typically associated with reduced energetic investment in maintenance and growth, and increased investment in reproduction and defence.

Our review has assumed that these life history transitions were primarily driven by plastic responses, and we have drawn on similar evidence from contemporary humans to provide mechanistic support. However, early agriculturalists may have replaced foragers in any given niche, as well as exposing themselves to new selective pressures, hence genetic factors undoubtedly merit further research. The population growth that followed the transition to agriculture increased the opportunity for new mutations to manifest (255), while niche construction is likely to have intensified selection on certain genes (9, 256). In Table 4, we provide examples of genetic change in traits relevant to all four life history functions, likely to have occurred in response to selective pressures provoked by the transition to agriculture.

**Table 4:** Hypothesised selective pressures and genetic change impacting life history functions associated with the transition to agriculture.

**Inferred selective pressure**	**Change in alleles**	**Life history functions affected**	**References**
Increased burden of infectious disease	Most adaptations targeting coding variation related to human innate immune function have occurred in the last 6,000–13,000 years	Defence	(257)
Rising population density and pools of standing water favour mosquito-borne diseases	Selection for various forms of haemoglobinopathy in last 5,000 years, providing protection against malaria	Defence	(258)
Changes in the physical properties of food	Quantitative genetic models show directional changes in skull morphology indicating trend toward lower masticatory demands	Growth	(259)
Domestication of plants	Modifications to Cytochrome P450 and NAT2 genes promote detoxification of plant secondary compounds	Defence	(256)
Introduction of dairying	Independent emergence of alleles for lactase persistence emerged in several different global regions in association with dairying	Growth	(260)
Increased consumption of starch from new crops	Genetic variants of TCF7L2 associated with improved blood sugar regulation evolved in three global regions at the same time as agricultural transition	Maintenance	(261)
Consumption of new fermented products that contain alcohol	Selection on alcohol dehydrogenase alleles resulting in more efficient ethanol metabolism	Maintenance	(262)
Increased risk of famine associated with crop failure	Derived GIP-1920A haplotype could have maintained higher maternal blood sugar levels during famines, favouring fetal survival and growth	Reproduction	(263)

While these trade-offs seem to generally hold for most of the available evidence, we may expect variations in some populations dependent upon a variety of ecological factors including the nutritional composition of crops and the local infectious disease burden. In that sense, we suggest that “the exceptions prove the rule,” in that it is also possible for the adoption of agriculture to elicit different life history strategies through the same plastic mechanisms. For example, should farm yields and ecological conditions permit, greater energy might be allocated to growth. More broadly, our framework can also be applied to populations that did not adopt agriculture, including contemporary foraging societies, or those currently transitioning, as discussed in Box 2.

Box 2 **Populations that did not adopt agriculture.**

While our focus has been on the transition to agriculture, much may be gained from extending the investigation of trends in life history trade-offs to populations that did not adopt any kind of farming, or who made only transient shifts toward agricultural subsistence, or who are only just starting to make this transition.

In the long-term past, populations that continued to forage provide a key reference against which to compare early farmers. Prehistoric foragers did not necessarily inhabit stable ecological environments, and may for example have had to adapt to major climatic change, as highlighted by research on the Natufians in the Levant (2, 264). Moreover, populations that persisted in foraging may have been exposed to the impact of neighbouring farmers on the local ecology (9), and over longer time periods foragers were increasingly pushed toward more marginal habitats (191).

Similarly, it is possible to study more recent “transitions to agriculture,” where foraging is only recently or currently being abandoned. Examples include the Toba and Wichí of the Argentine Gran Chaco (265), the Tsimane in Bolivia (266), the Pume in Venezuela (267), the Ache in Paraguay (268), and the Hadza in Tanzania (269). Other researchers have addressed this opportunity by studying groups of farmers and foragers that are closely related, such as the Bofi of the Central African Republic (270).

Such research can provide unique insight into the shifting trade-offs that we consider fundamental to the transition in the past. For example, a study of the Agta, a foraging population from the Philippines, found that more sedentary groups engaging in horticulture demonstrated increased levels of viral and helminthic infections but also higher fertility levels compared to those still foraging, thus supporting the notion that the shift toward sedentary life diverts energy toward defence and reproduction (271).

### The Central Role of Women and Inter-Generational Effects

While life history trade-offs could have emerged both through genetic adaptation, and life-course plasticity, it is worth focusing briefly on inter-generational trade-offs. The transition to agriculture had major impact on women, for several reasons. First, as highlighted above, increases in fertility inherently place unique energetic stresses on women, through the processes of pregnancy and lactation. While agriculture made possible new cereal-based complementary foods, allowing populations to wean their offspring earlier than typical of foragers (272), the changes may also have accelerated the rate at which successive offspring were produced. Second, women's subsistence tasks also changed. There is strong evidence that women performed a high proportion of repetitive subsistence-related labour, following the adoption of agriculture in central Europe. In particular, habitual loading of the upper limbs due to repetitive use of the saddle quern to process grain, led women to have greater mechanical loading than contemporary athletes (273). This labour may have simultaneously raised their energy needs, whilst also increasing their exposure to pathogens. While much of the evidence suggests a decrease in terrestrial mobility associated with the transition to agriculture in most but not all contexts (222, 246, 274, 275), this may have been counterbalanced by an increase in manual labour among both sexes (273, 276), so specific aspects of behavioural shifts associated with the transition to agriculture are expected to be spatially and temporally variable (277).

**Figure 7:**
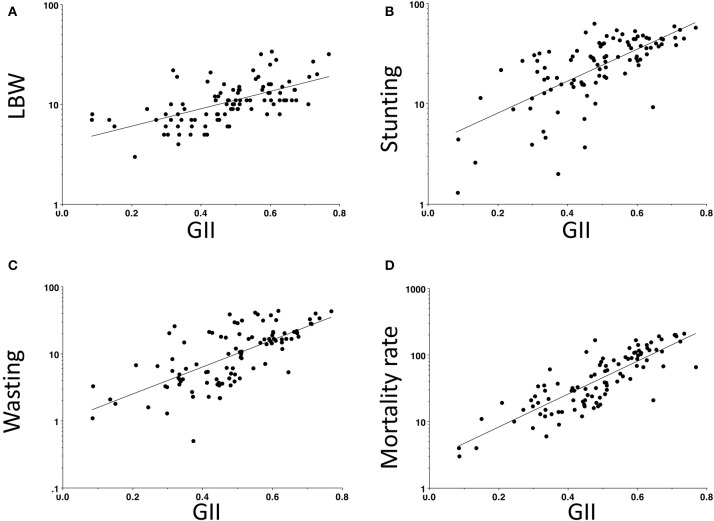
Associations of the Gender Inequality Index (GII), a marker of societal gender inequality, with the prevalence of **(A)** low birth weight, **(B)** child stunting, **(C)** child wasting, and **(D)** the risk of child mortality in the first 5 years of life, across 96 countries. Reproduced with permission from Marphatia et al. (278).

The notion that energetic stresses experienced by women propagate metabolic penalties to the next generation is supported by data on contemporary human populations. For example, across 96 countries, an index of societal gender inequality (indicating women's low status in society relative to men, mediated by a lack of access to resources and opportunities that promote health, education, and autonomy) was associated with three markers of child under-nutrition (low birth weight, and child stunting and wasting) as well as the risk of child mortality in the first 5 years of life (Figure 7) (278). In contemporary populations, women continue to be allocated both subsistence tasks as well as the primary responsibility for looking after infants and young children.

However, many studies have shown that male offspring are more susceptible to malnutrition in early life (279), most likely because their faster growth rate makes them more sensitive to any constraints on energy supply. Of interest here, there is evidence for more significant body size shifts among men than women (220), which suggests that male offspring disproportionately picked up the signal of energetic stresses affecting adult women.

### Unanswered Questions

While we have found supportive evidence for our primary hypothesis, that the adoption of agriculture profoundly changed human biology through re-organising life history trade-offs, many more specific questions remain. Given the considerable spatial, temporal, ecological, and cultural variation in the transition to agriculture globally, one would not predict a uniform response in different regions. Our key aim at this stage has been to provide a broad and solid conceptual framework that may inform and guide such future research questions. A series of issues meriting further work, regarding the timing of change, the environmental factors responsible, and the biological mechanisms involved, are listed in Table 5.

**Table 5:** Issues that merit investigation in future work.

**Timing** Which periods generated the greatest selective pressures, opportunities or stresses, and drove the most marked life history shifts? How correlated were life history trade-offs temporally? What were the implications for human life-history trade-offs of first domesticating crops vs. animals? Were there periods that favoured increased energy allocation to growth and maintenance? How did past disease epidemics emerge, and in which periods did mortality risk peak? **Environment** How did human life-history trade-offs vary in association with different types of agriculture? How did trade-offs vary in association with different ecological conditions? To what extent did “labour traps” associated with dietary and cultural transitions determine energetic allocation toward activity? How did changing activity patterns shift energy allocation through the lifespan? **Mechanism** To what extent were genetic vs. plastic responses involved? Beyond energy supply, what other “nutritional currencies”—e.g., macronutrients/micronutrients—drove trade-offs? What was the shape of life history trade-offs (linear, non-linear)? Were trade-offs conditional on phenotype, or on developmental experience?

Progress in investigating these questions requires more integrative approaches to the bioarchaeology of past populations. Research programmes in this field are often determined by focus and methodology, investigating variation in prehistoric human health, diet, or activity in isolation. Studies that are beginning to combine relevant datasets in the study of prehistoric dietary transitions, incorporating for example the study of body size, activity patterns, and diet (280), provide a model of such fruitful integration. Major global comparisons of prehistoric health, such as those conducted in the “Global History of Human Health” project (240, 281, 282), provide useful integration of relevant palaeopathological and growth data, but would benefit from broader integration and theoretical context to begin to investigate past life history transitions.

A key challenge for bioarchaeologists is the interpretation of detailed demographic and life history data from skeletal assemblages. There are many approaches to palaeodemographic interpretation of factors relevant to the interpretation of life history traits, such as population structure, mortality, and migration (283), the challenges of which have been discussed at length (284). New osteological approaches have also been developed for the interpretation of fertility (285) and the timing of puberty (286) that deserve greater attention. Future research could address many of the questions posed above through systematic comparison of skeletal assemblages and the integration of bioarchaeological studies of prehistoric growth, activity, diet, and pathology with skeletal estimates of life history parameters including fertility, birth weight, age at menarche, and age at death and mortality profiles. There are also opportunities to apply modelling approaches. For example, both human biology and agriculture can be approached through the lens of “risk management” (287, 288).

For those addressing genetic adaptations, a current limitation is the bias of genome-wide association (GWA) studies toward individuals of European ancestry. For example, a summary of GWA studies reported up to 2019 found that 78.4% of individuals included in such studies were of European ancestry, and just 10.2, 2.0, and 1.3% of Asian, African or Hispanic/Latin American ancestry, respectively (289). Further work could provide a more comprehensive perspective on genetic change associated with the transition to agriculture.

While our main aim is to encourage application of the life history theoretical framework to the archaeological record, it may also be used to shed light on life history traits in contemporary farmers, especially where they have practiced a specific form of agriculture for many centuries (Box 3). One intriguing issue relates to human—plant—parasite interactions. Although cultivated crops most obviously supply human energy needs, they may also supply specific nutrients that promote immune defence against local pathogens (299). For example, the cultivation of fava beans is common among circum-Mediterranean populations, and dates back to ~8,500 years in the Levant. These populations also demonstrate high levels of deficiency in the enzyme glucose-6-phosphate dehydrogenase (G6PD), and both G6PD deficiency and fava beans increase risk of “favism,” a form of acute haemolytic anaemia. However, G6PD deficiency also confers protection against malaria, and this protection is enhanced by consumption of fava beans (299). This and other examples indicate that the type of crops cultivated could alter the impact of pathogens on human biology, with potential implications for life history trade-offs.

Box 3 **Life history adaptations evident in contemporary farmers.**

One example of how a particular form of agriculture has left a signal in contemporary life history trade-offs is given by the Sardinian population, a genetic isolate occupying an island off the Italian mainland. Their subsistence mode was historically based on sheep farming and cultivating cereals and legumes, under the notable ecological stress of endemic malaria. Until recently, the typical phenotype of Sardinians included short stature (290) but also longevity, indicated by a high prevalence of centenarians (291), as well as lactose intolerance (292). The population also shows a very high prevalence of G6DP deficiency, which can be attributed to the selective pressure of malaria. Co-adaptation of the microbiota also appears to contribute to longevity (291), whereas gene polymorphisms of cytokines playing a major regulatory role in the inflammatory response are not associated with life expectancy (293). The microbiome can impact many metabolic traits in the host, for example by varying in its species diversity, the presence of species that aid the digestion of particular diets, and its inflammatory profile (294–296). This suggests that, aside from any selective pressures acting directly on human genetic determinants of lifespan, the transition to agriculture might also have elicited life history trade-offs through changes in the genetic profile of the microbiome.

In recent decades, the eradication of malaria, nutrition transition, and dietary change has elicited a rapid secular trend in height in Sardinia, greater than elsewhere in Italy (290), but also increased rates of auto-immune diseases such as coeliac disease and type 1 diabetes (292, 297, 298). The high levels of these diseases may reflect the overloading of homeostatic traits that evolved to optimise fitness in pre-modern conditions.

Overall, we hope that our conceptual approach will stimulate more work on the transition to agriculture, and indeed it could also be applied to other transformations of the human subsistence niche, as briefly reviewed next.

## Beyond Agriculture

The life history transitions that we have focused on around the origins of agriculture are by no means unique. Our over-arching hypothesis is that much adaptive change in humans may be underpinned by such life history transitions. There is evidence that the trends we discussed above were already operating at slower paces during the palaeolithic, and we can project them back into the deeper past. Indeed, contrasting with the current focus on skeletal traits such as the form of bipedalism and the size of the adult brain, the entire evolutionary history of hominins can be portrayed as the evolution of different life history strategies, as explored in another paper in this collection (300). The same approach can also be used to reconstruct the evolution of human childhood and “emerging adulthood” (301, 302).

**Figure 8:**
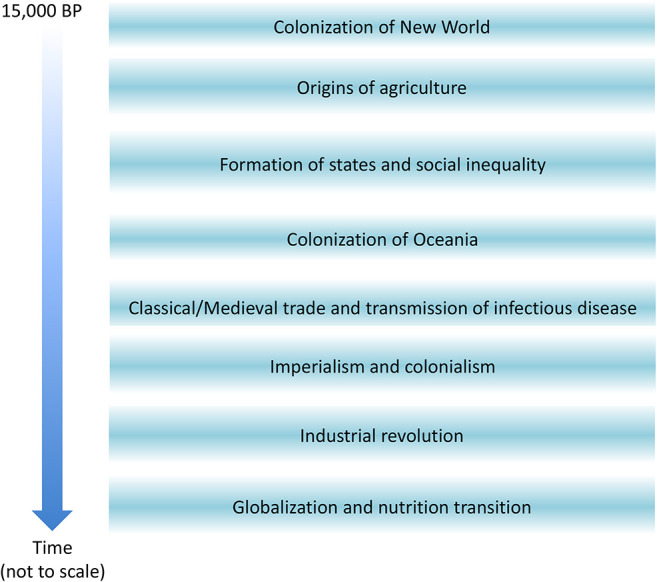
Potential events in human evolutionary and recent history, where changes in mortality risk and dietary subsistence may have elicited the reorganisation of human life history strategy.

Similar trade-offs are expected to have occurred since the origins of agriculture. Figure 8 summarises a series of events in recent human history where combined changes in mortality risk and subsistence niche can be expected to have elicited the reorganisation of human life history strategy. Some of these have already been supported by evidence. For example, Stock and Migliano linked a reduction in stature among Great Andamanese Islanders with increased mortality associated with exposure to British colonial rule (303). We briefly consider in more detail two recent examples.

### Onset of Industrialisation

The early industrial revolution was another period in which, paradoxically, substantial population growth occurred in the UK while markers of health and, in some populations, life expectancy declined. These correlated trends were highlighted in the nineteenth century by pioneering political economists, who understood very well that while the overall supply of food was increasing, many of the new factory workers were exposed to appalling living conditions and suffered high rates of infant, child, and adult morbidity and mortality (304).

Data on soldiers born in the southern part of the UK indicate a broad decline in adult height from the mid eighteenth to the mid- nineteenth century, reaching a nadir around 1,855 (305). At the same time, the rapidly growing industrial cities were characterised by worsening air pollution and exposure to infectious disease (304). Adults also demonstrated high levels of degenerative diseases, which were directly linked with poor living conditions (306). Nonetheless, the nineteenth century also saw substantial population growth in the UK, from around 11 million in 1,801 to 37 million by 1,901 (307).

These trends match closely with those we have described for agriculture, and indicate the diversion of energy to immune function and reproduction, at the expense of growth and maintenance. Another similarity is that these life history transitions occurred under the influence of dietary change, as new industrial foodstuffs (bread, jam) and imported foods from overseas colonies were used to reduce the costs of expanding the new urban proletariat (308).

### Nutrition Transition

The latest life history transition could be said to be taking place through globalisation and the nutrition transition. In high-income countries, the long-term transitions have been favourable to health, indicating the benefits of better food supplies and public health efforts to combat infectious disease (89). Industrialised countries have seen secular increases in height as well as steady improvements in life expectancy, and both of these have been directly associated with declines in infant mortality rate, indicating a lower allocation of energy to immune defence in early life (89). The twentieth century has also seen major demographic changes, encompassing both later onset of reproduction and reduced family size. These demographic changes have been in large part achieved by the uptake of various forms of contraception. Thus, in high-income countries, life history transitions have seen a re-allocation of energy to growth and maintenance, over reproduction, and defence.

In low- and middle-income countries, however, the trends are more complex. Secular increases in height have been relatively modest, especially in south Asia and sub-Saharan Africa (309), whereas increases in obesity have been much more noticeable (310). Improvements in life expectancy have been variable, and epidemics such as HIV briefly reduced it in some countries. Moreover, within recent decades, around 80% of the global burden of chronic non-communicable disease is now occurring in low- and middle-income countries (311).

Why are these trends different from those in high-income countries? A key factor is likely to be the persisting high burden of infectious disease, which is detrimental both to child growth and health (maintenance) (89), as well as other social and environmental stresses (312). Given higher extrinsic mortality risk, it is arguably unsurprising that energy allocation to growth and maintenance is constrained in favour of greater allocation to reproduction and defence. Contrasting with the modest secular increase in height, many populations are showing substantial increases in central abdominal fat, as well as secular declines in the average age at menarche (313). These trends may be exacerbated by the fact that nutrition transition is not only increasing energy availability, but also changing the composition of the diet, making it more obesogenic (314).

## Conclusions

In summary, we have used life history theory to consider how rapid environmental shifts may have impacted human growth and development by orchestrating coordinated and synchronic life-history trade-offs in human populations. The primary change appears to have been a systematic shift toward allocating energy to reproduction and defence, indicated by population growth and both direct and indirect indications of higher infectious disease load. This shift reduced the energy available for growth and maintenance, indicated by declines in stature and an increase in markers of degenerative bone disease. Where populations did not follow this general pattern, we can still use life history theory to understand how different life history transitions emerged.

The conceptual model that we developed may help understand other major transitions such as industrialisation and rapid nutrition transition. Over the last 150 years in high-income countries, public health efforts have simultaneously improved diet and reduced infection risk, thus reversing the life history transitions that were provoked by adopting agriculture (8). In contemporary low and middle income countries, conversely, where infectious disease burdens remain high for both infants/children and adults, and agricultural yields have been poor for decades, the subsistence niche has changed substantially less over centuries (though this is also related to historical trends such as colonialism) (8). As rapid nutrition transition occurs, the change in energy availability is not accompanied by equally rapid changes in broader living conditions, providing us with new insight into why the primary secular trends relate more to adiposity than to adult height.

We thus link the construction of novel niches with life history responses, including evolutionary strategies for body size. This approach may ultimately help understand how developmental plasticity mediates links between changes in our subsistence niche and human health outcomes.

## Author Contributions

JW conceived the original idea (10). JW and JS developed the idea in detail and co-wrote the manuscript.

## Conflict of Interest

The authors declare that the research was conducted in the absence of any commercial or financial relationships that could be construed as a potential conflict of interest.
